# Detection of *Fusobacterium nucleatum* in Colorectal Adenomas Reveals Associations with Immune Molecular Signatures

**DOI:** 10.3390/ijms27167258

**Published:** 2026-08-14

**Authors:** Sara Samir Foad Al-Badran, Natalie Fisher, Philip D. Dunne, Mark Johnstone, Noori Maka, William M. Rooney, Samantha Campbell, Paul Capewell, Ditte Andersen, Gerard Lynch, Stephen McSorley, Joanne Edwards

**Affiliations:** 1 Wolfson-Wohl Cancer Research Center, School of Cancer Sciences, University of Glasgow, Garscube Estate, Switchback Road, Bearsden G61 1QH, UK; mark.johnstone.2@glasgow.ac.uk (M.J.); gerard.lynch@glasgow.ac.uk (G.L.); stephen.mcsorley@glasgow.ac.uk (S.M.); 2 School of Medicine, Dentistry and Biomedical Sciences, Queen’s University Belfast, University Road, Belfast BT7 1NN, UKp.dunne@qub.ac.uk (P.D.D.); 3 CRUK Beatson Institute, Garscube Estate, Switchback Road, Bearsden G61 1BD, UK; 4 School of Medicine, Wolfson Medical School Building, University of Glasgow, University Avenue, Glasgow G12 8QQ, UK; 5 NHS Greater Glasgow & Clyde, 1055 Great Western Road, Glasgow G12 0XH, UK; noori.maka2@nhs.scot; 6 BioClavis Ltd., Clydebank, 201 Dumbarton Road, Glasgow G81 4XJ, UK; will.rooney@glasgow.ac.uk (W.M.R.); s.campbell@crukscotlandinstitute.ac.uk (S.C.); paul.capewell@glasgow.ac.uk (P.C.);

**Keywords:** *Fusobacterium nucleatum*, microbiome, adenoma, colorectal cancer, mutational signature, transcriptomics, bowel screening

## Abstract

*Fusobacterium nucleatum* has been implicated in colorectal cancer, but its role in adenomas remains unclear. We applied an RNA-based detection of *F. nucleatum* in formalin-fixed paraffin-embedded adenoma tissue and explored the mutational landscape and transcriptomic profile of *F. nucleatum*+ patients in comparison to *F. nucleatum*- patients. Bespoke *F. nucleatum* probes successfully detected *F. nucleatum* in 11% of adenomas. *F. nucleatum*+ patients exhibited a positively enriched anti-bacterial defence response and immune-related transcriptomic signatures, as well as a proliferative profile. These findings suggest that *F. nucleatum* positivity is associated with immune and proliferative transcriptomic signatures in colorectal adenomas, but this requires validation in larger, longitudinal cohorts.

## 1. Introduction

As the third most commonly diagnosed cancer worldwide [1], the pre-malignant origins of colorectal cancer (CRC), known as colorectal adenomas, carry clinical relevance. Since the progression to malignancy can take between 7 and 15 years, detection and removal of polyps is a major advantage of CRC screening programmes. However, the removal of an adenoma by polypectomy does not eradicate the risk of further adenomas, and so the British Society of Gastroenterology (BSG) has guidelines for post-polypectomy surveillance to stratify patients at higher risk of future (metachronous) adenomas to develop [2]. These guidelines depend on the size, number, and cellular atypia present in the removed tissue, and while these are important properties, they are not comprehensive of the mechanisms that drive growth.

The microbiome, referring to the various bacterial, viral, and fungal residents of the human gut, has long been implicated in both disease and health, with the gut microbiome being heavily studied in CRC research, with various microbiome-centred CRC-driving effects like genotoxicity, immune modulatory capacity, and metabolic effects being reported [3]. *Fusobacterium nucleatum* is a Gram-negative, rod-shaped, anaerobic bacterium that has been implicated in various diseases, including CRC. Small-scale sequencing studies have found that Fusobacterium species were enriched not only in CRC tissue [4,5] but also in colorectal adenoma tissue and in stool samples from individuals with colorectal adenomas or CRC, when compared to matched normal samples [6]. Higher *F. nucleatum* levels have been associated with poor cancer-specific survival, poor prognostic factors including right-sided cancers, poorly differentiated cancers, and high CpG island methylator phenotype status [7]. *F. nucleatum* was also found in CRC liver metastases and was associated with the presence of tumour cells at distant sites [8].

The role of *F. nucleatum* in CRC development and progression has been elucidated through mechanistic studies, and although involvement with colorectal adenomas has been reported [6,9,10,11], its role in metachronous adenoma development is not well understood. In this exploratory study, we hypothesised that the presence of *F. nucleatum* in adenomas is associated with distinct molecular signatures that may influence the development of metachronous colorectal adenomas in bowel screening patients.

## 2. Results

### 2.1. Cohort Description

**Figure 1:**
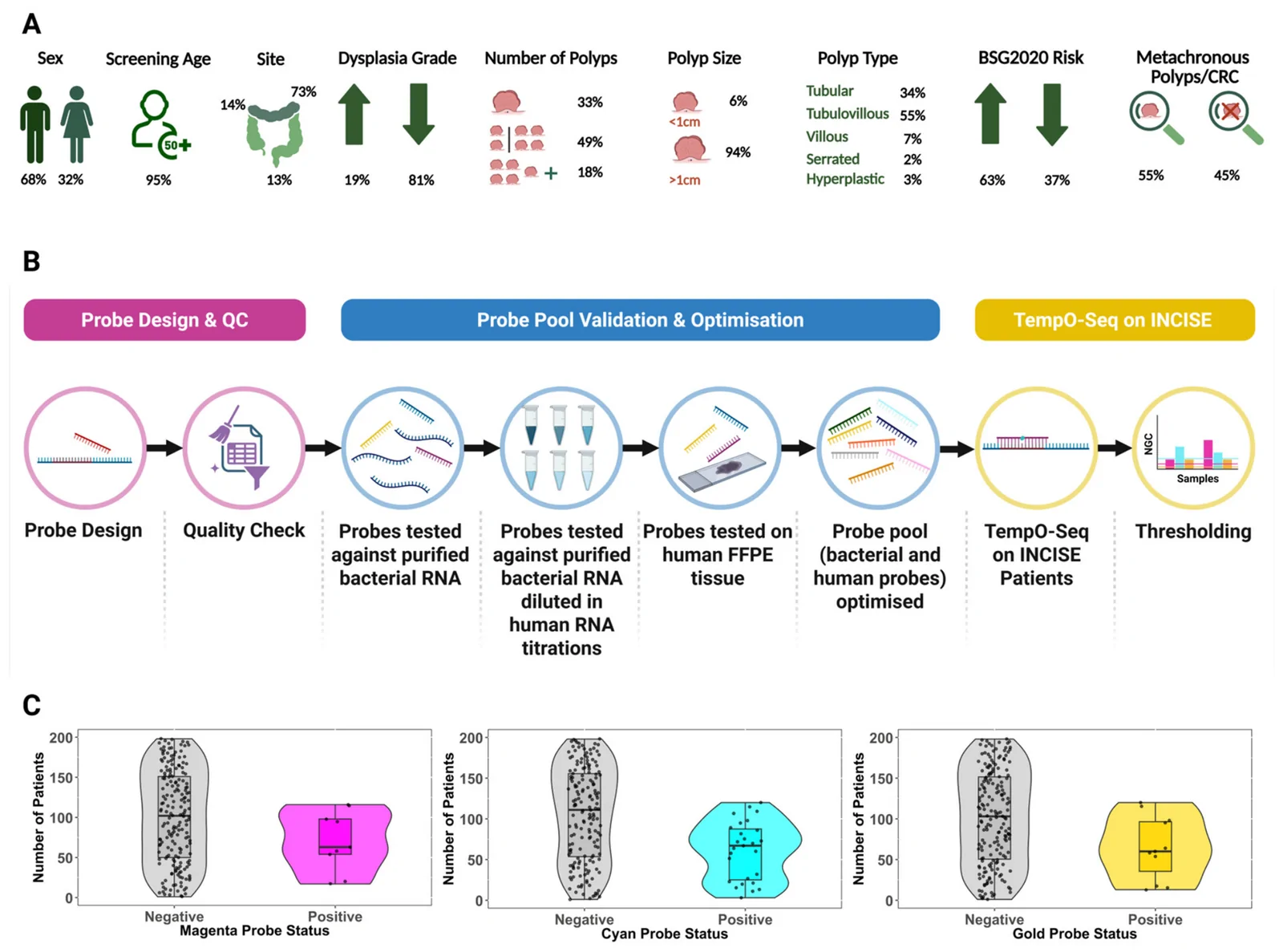
Bespoke TempO-Seq™ Probes Detect *F. nucleatum* RNA in Human Adenoma FFPE Tissue. (**A**) Description of the *F. nucleatum* INCISE Cohort in relation to patient clinical features. (**B**) Schematic of workflow of bespoke probe design, validation, and optimisation of probe pool. (**C**) Distribution of probe detection of *F. nucleatum*+ samples for the three selected probes, magenta (**left**), cyan (**middle**), and gold (**right**).

This cohort consisted of full-thickness tissue from 198 INCISE patients. 68% of patients were male (n = 134), 95% were of screening age (n = 188), and 73% of patients presented with polyps in the left colon (n = 145). 19% of patients had polyps with high-grade dysplasia (n = 37), 49% of patients presented with 2–4 polyps (n = 96), 94% had polyps larger than 10 mm (n = 187), and 55% of patients had polyps with tubulovillous features (n = 108). 63% of patients were found to be high risk according to current BSG2020 Guidelines (n = 125), and 55% of patients (n = 109) had metachronous polyps or CRC at follow-up (Figure 1A).

### 2.2. TempO-Seq™ Probe Validation and Detection Rate

**Figure A1:**
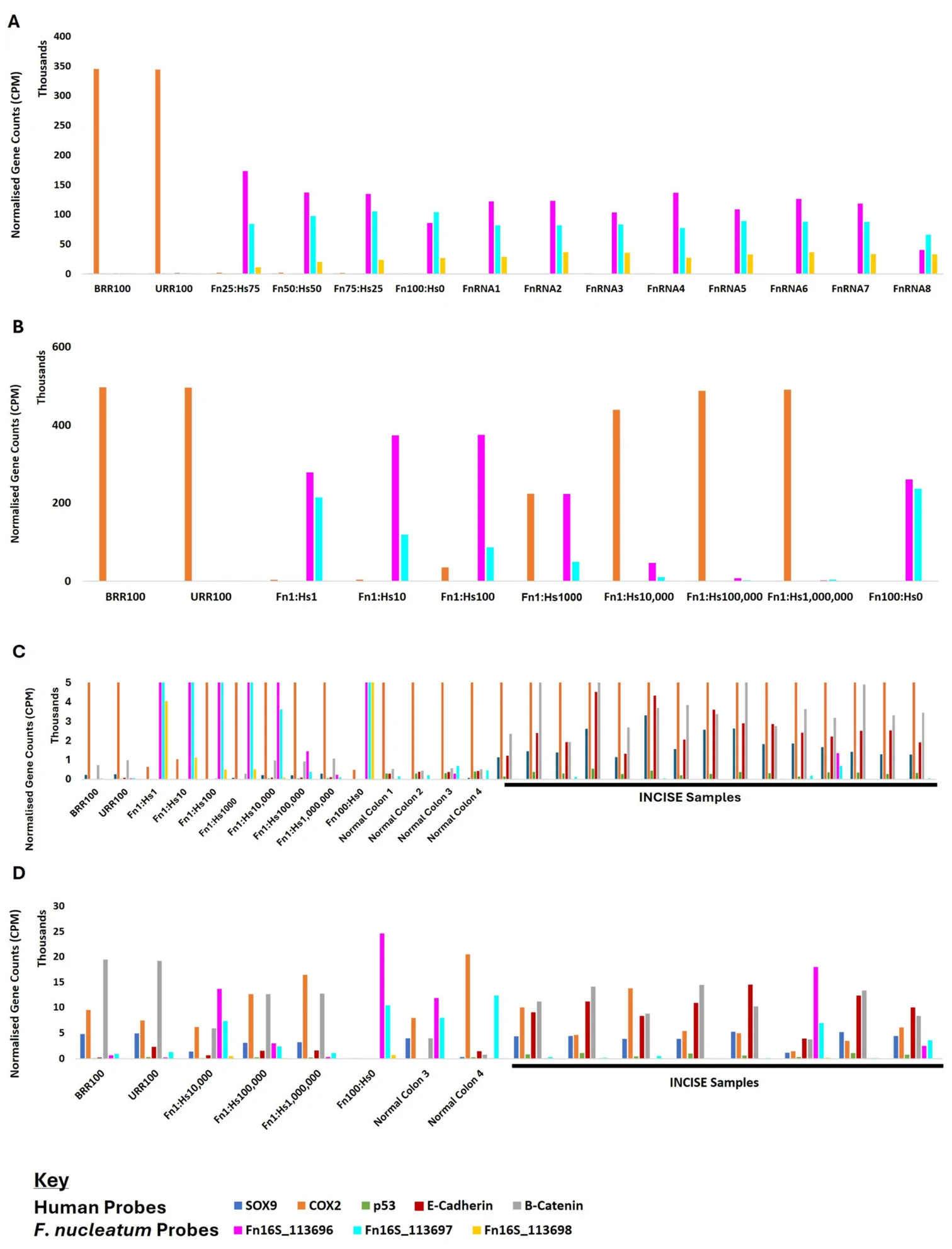
TempO-Seq™ Probes Quality Check and Pool Optimisation. (**A**) The signal from the human probes was mostly detected in the human reference RNA samples, with the COX2 probe dominating the majority of the reads. The *F. nucleatum* probes all generated a signal in the purified bacteria-human RNA mixed samples, the *F. nucleatum* RNA to human RNA samples, and in multiple replicates of *F. nucleatum*-only RNA, with the magenta probe providing the strongest signal in most of the samples, followed by the cyan and gold probes. The signal from the COX2 probe was also detected in the bacteria-human RNA mixed samples, with the signal levels gradually decreasing with decreasing human RNA concentrations. In the *F. nucleatum-only* RNA samples, no human probe signal was detected. (**B**) 10-fold serial dilutions of *F. nucleatum* RNA in human URR RNA used to test probe performance against a high background of human RNA. The bacterial probes were able to detect their targets in dilutions as high as 1:1 × 10^4^ (bacteria to human). Only human probes carried a detectable signal in the human reference RNA samples, while only bacterial probes were detectable in the pure *F. nucleatum* RNA samples. COX2 remained the most prominent human signal, and it continued to compress signals from other probes. (**C**) FFPE tissue lysates from healthy colon samples and lysates from 15 INCISE patients were tested alongside the serial dilution of bacterial-to-human RNA. The signal from the COX2 human probe continued to dominate most of the reads, while the 10-fold serial dilution controls exhibited results similar to previous observations, where the magenta and cyan probes could detect their targets in bacteria-to-human RNA dilutions as high as 1:1 × 10^4^. (**D**) COX2 probe signal repeatedly overpowered the signals from the other human probes the probe pool was optimised to allow signals from the remainder of the probes to be displayed more clearly. The COX2 probe had to be diluted to be 1% of the DO pool (0.03 nM in 3 nM of other human and bacterial probes) in the detector oligo (DO) pool.

**Figure A2:**
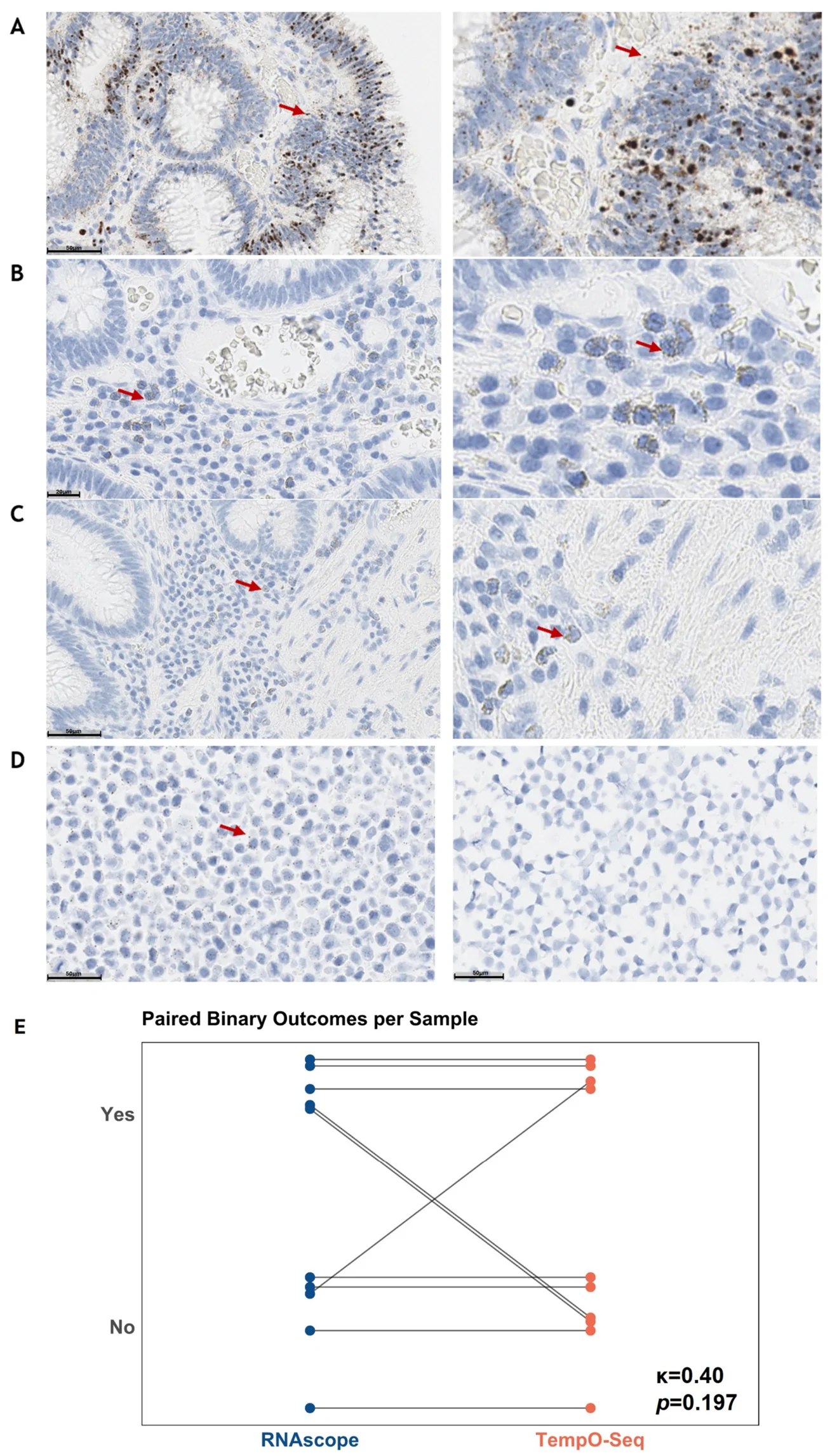
RNAscope^®^ as a Spatial Method to Validate the Presence of *F. nucleatum* RNA on Archival Human Adenoma Tissue from an INCISE Patient. (**A**: **Left**) HS-UBC universal probe for chromogenic RNAscope^®^. (**A**: **Right**) Zoomed in area from (**A**: **Left)**. (**B: Left**) 16S RNA bacterial probe for chromogenic RNAscope^®^. (**B**: **Right**) Zoomed in area from (**B**: **Left)**. (**C**: **Left**) 23S RNA *F. nucleatum* probe for chromogenic RNAscope^®^. (**C**: **Right**) Zoomed in area from (**C**: **Left)**. (**D**: **Left**) Positive Control: HS-TBP probe for chromogenic RNAscope^®^ on HeLa cell pellets (supplied by ACD, a biotechne company). (**D**: **Right**) Negative Control: DapB probe for chromogenic RNAscope^®^ on HeLa cell pellets (supplied by ACD, a biotechne company). (**General Key**): Brown dots are positive RNAscope^®^ signal. Larger brown dots indicate mRNA aggregates. Red arrows point at positive RNAscope^®^ signal. Scale bars denote 50 μm. (**E**) Concordance Plot between patients who were *F. nucleatum*+ in RNAscope^®^ and TempO-Seq™. RNAscope^®^ was carried out on samples from 10 patients.

**Figure A3:**
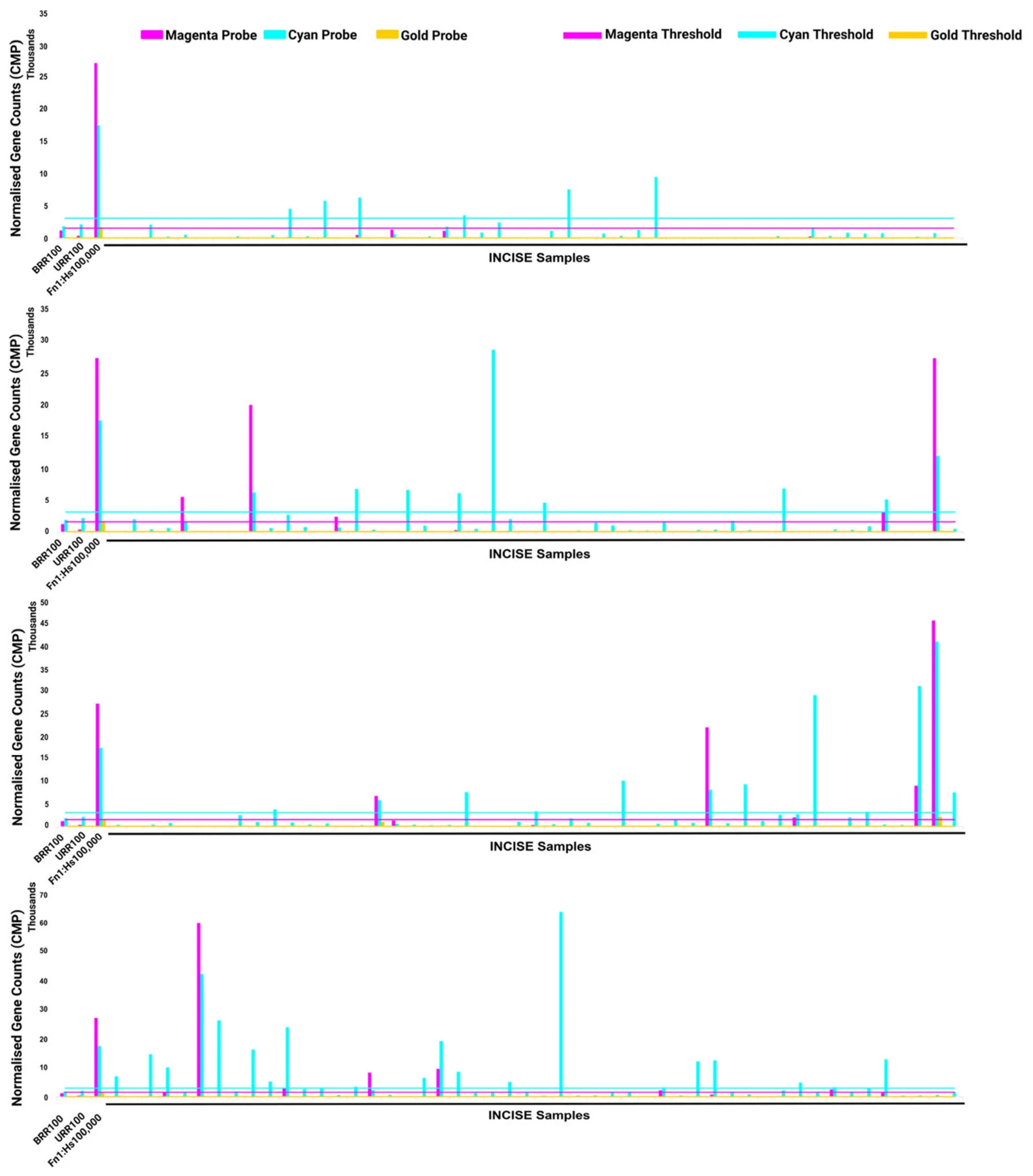
TempO-Seq™ on 198 INCISE patients. All samples with signal above the threshold for each probe (represented by a horizontal line of the same colour as the corresponding probe) were considered positive for that probe. Patients who were positive for at least two probes (any) were considered *F. nucleatum*+.

To ensure the bespoke *F. nucleatum* probes were specific and sensitive to their targets, we performed TempO-Seq™ on purified human reference RNA, purified *F. nucleatum* RNA, and a mixture of human and *F. nucleatum* RNA (Figure 1B, Figure A1). After validating the designed probes’ specificity and sensitivity to their targets, we sought to validate the specificity and sensitivity of *TempO*-Seq™ for *F. nucleatum* using the spatial assay RNAscope^®^ in situ hybridisation using *F. nucleatum* probes for the RNAscope^®^ assay, as an independent orthogonal assay on tissue from the same blocks used for TempO-Seq™ (Figure A2). Although concordance reflected reduced agreement (Figure A2E, κ-value = 0.4) as RNAscope^®^ was carried out on only 10 samples, TempO-Seq™ was performed on 198 INCISE lysates to increase output, using the optimised DO pool, with a read depth of 1588 counts per sample. All human probes were detectable, to varying degrees (Figure 1C), suggesting that the TempO-Seq™ run was successful. The three *F. nucleatum* probes were detectable in the bacterial-human RNA control and displayed strong signals in some of the INCISE lysates. To determine which samples were to be considered positive and negative for *F. nucleatum* (*F. nucleatum*+ and *F. nucleatum*-, respectively), thresholds (Table 1; Figure A3) were calculated for each bacterial probe from the averaged read counts plus the standard deviation of the human reference RNA controls.

Samples that were above the threshold for at least two probes were considered *F. nucleatum*+. 6% (n = 12) of samples were positive for any two probes, and 5% (n = 10) of samples were above the threshold for all three probes, with a total positive group of 11% (n = 22).

The correlations between the *F. nucleatum*+ and *F. nucleatum*- patients and clinical factors were investigated using chi-squared analysis (Table A5) to reveal a significant correlation between *F. nucleatum*+ status and non-adenomatous polyps (*p* = 0.003). However, *F. nucleatum*+ status was not significant for Cox regression analysis (Table A6).

### 2.3. Mutational Profiling of F. nucleatum+ Patients

**Figure 2:**
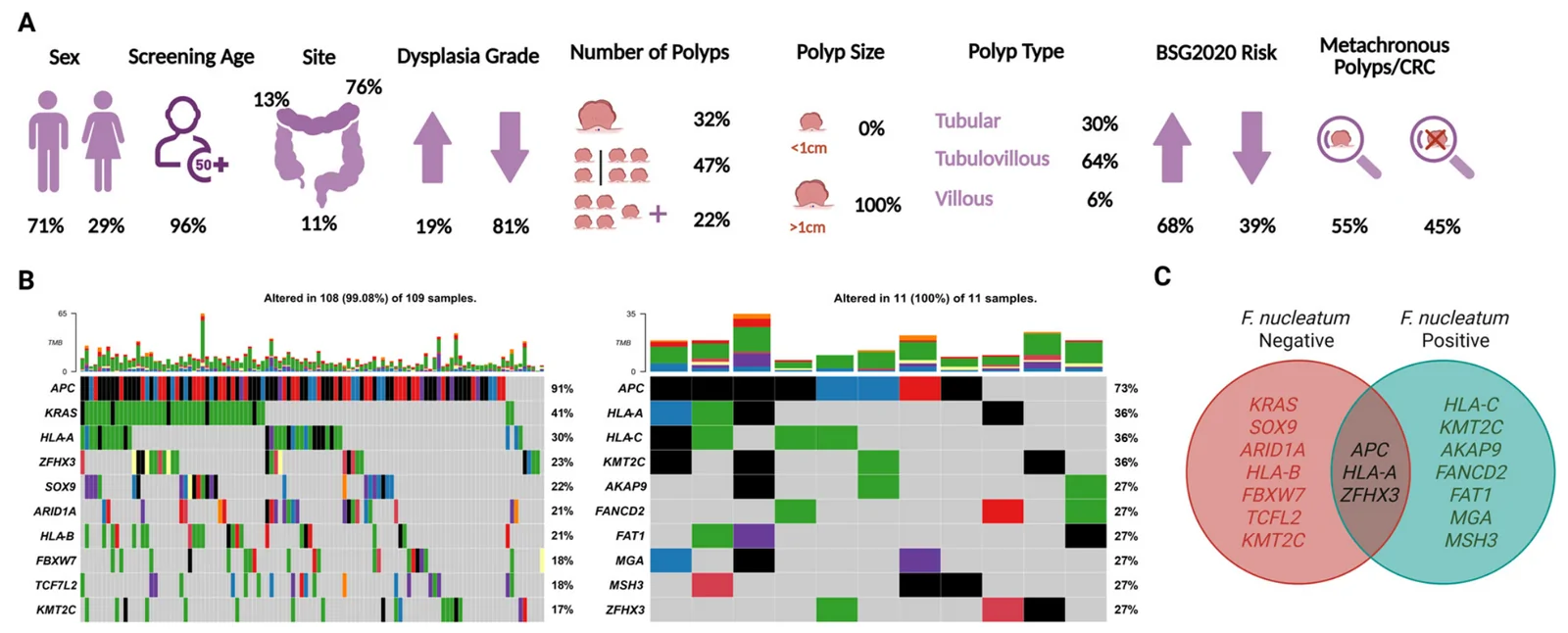
Descriptive Analysis of Mutational Landscape Suggests Differences between *F. nucleatum*+ and *F. nucleatum*- Adenoma Patients. (**A**) Description of the *F. nucleatum* INCISE Cohort with mutational data available in relation to patient clinical features. (**B**) Oncoplots of *F. nucleatum*- patients (**left**) and *F. nucleatum*+ patients (**right**), showcasing the top 10 mutated genes in each group ranked by percentage of samples affected for each gene, and then alphabetically by gene name. Colours indicated the mutations as red for nonsense, green for missense, dark blue for frameshift deletion, purple for frameshift insertion, yellow for in-frame deletion, pink for in-frame insertion, light blue for nonsense mutation, orange for splice sites, and black for multi-hit mutations. (**C**) Venn diagram of host-driven mutated genes that overlap between the two groups.

Next, DNA from adenomas from 120 INCISE patients who had TempO-Seq™-guided *F. nucleatum* status underwent genomic sequencing and analysis to reveal a difference between the mutational profiles of *F. nucleatum*+ and *F. nucleatum*- patients. 71% of patients were male (n = 86), 96% were of screening age (n = 115), and 76% of patients presented with polyps in the left colon (n = 91). 19% of patients had polyps with high-grade dysplasia (n = 23), 47% of patients presented with 2–4 polyps (n = 56), 100% had polyps larger than 10 mm (n = 120), and 64% of patients had polyps with tubulovillous features (n = 77). 68% of patients were found to be high risk according to current BSG2020 Guidelines (n = 82), and 55% of patients (n = 66) had metachronous polyps or CRC at follow-up (Figure 2A).

Of the *F. nucleatum* patients with mutational data available, 99% (n = 108) had mutations (Figure 2B). *APC* (91%) and *KRAS* (41%) were the top two most mutated genes with “multi-hit” and “missense” mutations, respectively.

Although 100% of *F. nucleatum*+ patients with mutational data available had mutations, the number of patients in this subset is very low (n = 11). This led to some different mutational trends than previously described. Although *APC* is still the top mutated gene (73%) with mainly multi-hit events, *KRAS* was no longer among the top ten most mutated genes (Figure 2B). Three genes overlapped between *F. nucleatum*+ and *F. nucleatum*- patients: *APC*, *HLA-A*, and *ZFHX3* (Figure 2C).

### 2.4. Bulk Transcriptomics Analysis of F. nucleatum+ Patients

Although numbers were low for the *F. nucleatum*+ patients for which mutational data were available, the mutations seen in immune-related genes like *HLA-A* and *HLA-B*, and the transcriptomic profile of these patients were of interest to investigate the biological processes underlying the host-bacterial interaction.

**Figure 3:**
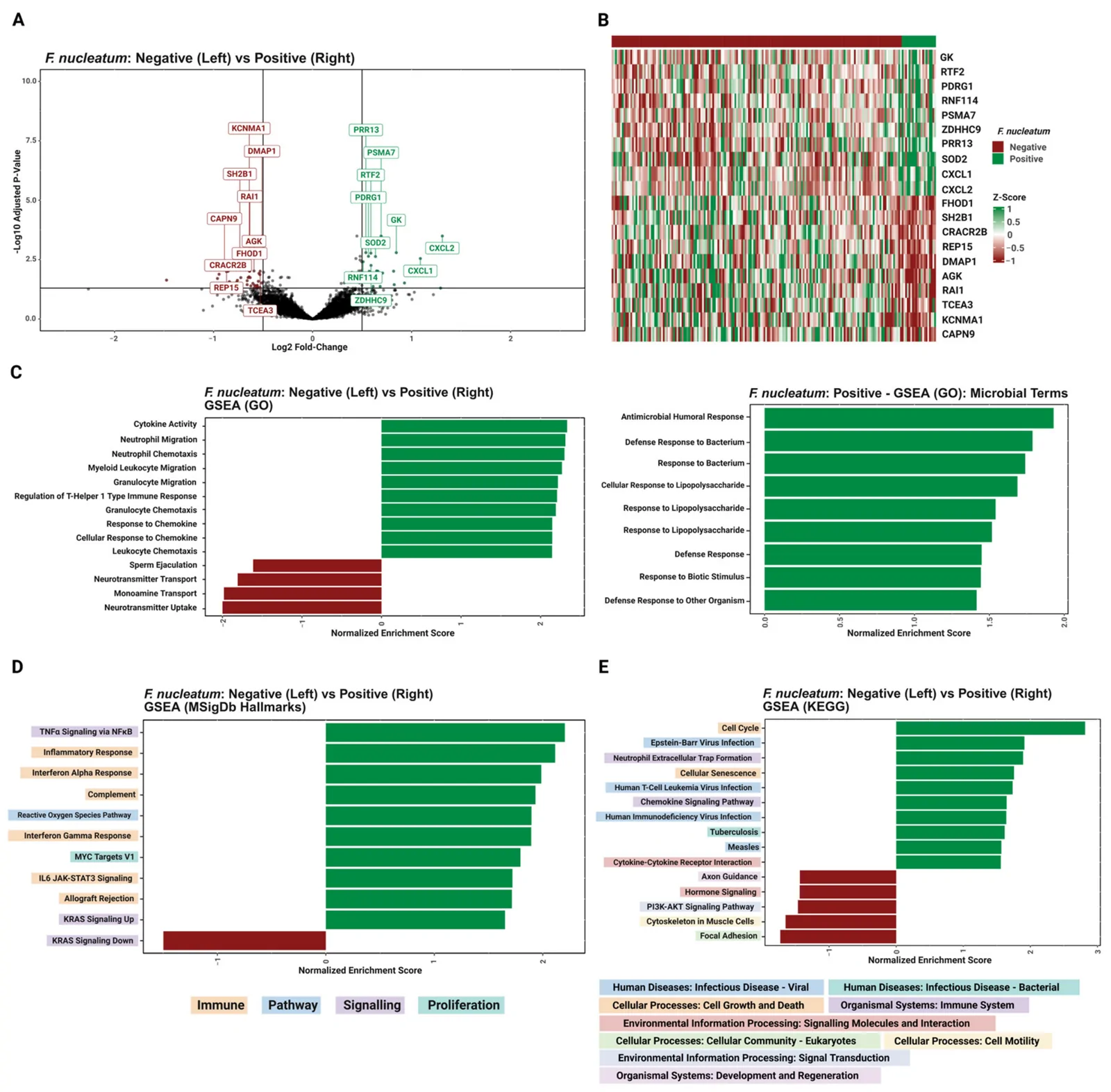
Transcriptomic Profiling Reveals Immune and Proliferative Signatures in *F. nucleatum*+ Adenoma Patients. (**A**) Volcano plot of differentially expressed genes (DEGs) in *F. nucleatum*+ patients. Thresholding used *p*Adj < 0.05 and Log2 Fold-Change of <−0.5 and >0.5. Green genes are overexpressed in *F. nucleatum*+ patients, while red genes are underexpressed. (**B**) Heatmap of the DEGs highlighted in the volcano plot with a z-score indicator ranging from −1 for underexpression to 1 for overexpression. (**C**) Gene set enrichment analysis using Gene Ontology terms. Thresholding was set at a *p*Adj < 0.05 and a normalised enrichment score (NES) <−1 or >1. The top 10 positively enriched (green) and negatively enriched (red) terms in *F. nucleatum*+ patients are shown on the **left**, while bacteria-centred positively enriched terms are shown on the **right**. (**D**) Gene set enrichment analysis using Molecular Signalling Database gene sets. Thresholding was set at a *p*Adj < 0.05 and a normalised enrichment score (NES) <−1 or >1. The top 10 positively enriched (green) and negatively enriched (red) gene sets in *F. nucleatum*+ patients are shown, with each gene set highlighted to correspond to its category. (**E**) Gene set enrichment analysis using the Kyoto Encyclopedia of Genes and Genomes. Thresholding was set at a *p*Adj < 0.05 and a normalised enrichment score (NES) <−1 or >1. The top 10 positively enriched (green) and negatively enriched (red) pathways in *F. nucleatum*+ patients are shown, with each pathway highlighted to correspond to its classification.

Of the 198 patients with TempO-Seq™-based *F. nucleatum* status, bulk transcriptomic data were available for 190 of them, all of which were included in the analysis. The patients were grouped into *F. nucleatum*- (n = 170 with bulk transcriptomics available) and *F. nucleatum*+ (n = 20 with bulk transcriptomics available). The *F. nucleatum*+ group was compared to the *F. nucleatum*- group (Figure 3A).

To assess the transcriptomic differences between the *F. nucleatum*- and *F. nucleatum*+ patients, DESeq analysis was used by applying the cut-off criteria of *p*Adj < 0.05 and Log2 Fold-Change of <−0.5 and >0.5. This revealed 29 upregulated genes and 38 downregulated genes, for a total of 67 differentially expressed genes, in the *F. nucleatum*+ group. The top 10 of each are displayed in Figure 3A. To visualise the expression pattern of each DEG across the two groups, a heatmap was generated, revealing a pattern consistent with the regulation direction observed in the volcano plot, which was verified by a heatmap focussing only on the top 10 upregulated and downregulated genes (Figure 3B).

To understand the molecular signatures enriched within the *F. nucleatum*+ samples, pairwise GSEA was performed using three publicly available biological resources: GO, MSigDb, and KEGG. Thresholding was set at a *p*Adj < 0.05 and a normalised enrichment score (NES) <−1 or >1. The top 10 positively and negatively enriched GO terms, ordered by the NES (Figure 3C), show a positive enrichment in immunity terms. Further analysis revealed multiple positively enriched terms that met the cut-off criteria but ranked below the top 10, which, interestingly, confer a signature of microbial infection recognition and response.

As MSigDb offers gene sets from multiple organisms, the Hallmarks human gene set collection was used to focus the analysis. Twenty-two gene sets had a *p*Adj < 0.05. Figure 3D shows the top ten activated and suppressed gene sets. Interestingly, of the top ten activated gene sets, six were categorised as “immune”, three were categorised as “signalling”, and one was in each of the “pathway” and “proliferation” categories, suggesting an immune-rich signature within the *F. nucleatum*+ patients.

**Figure A4:**
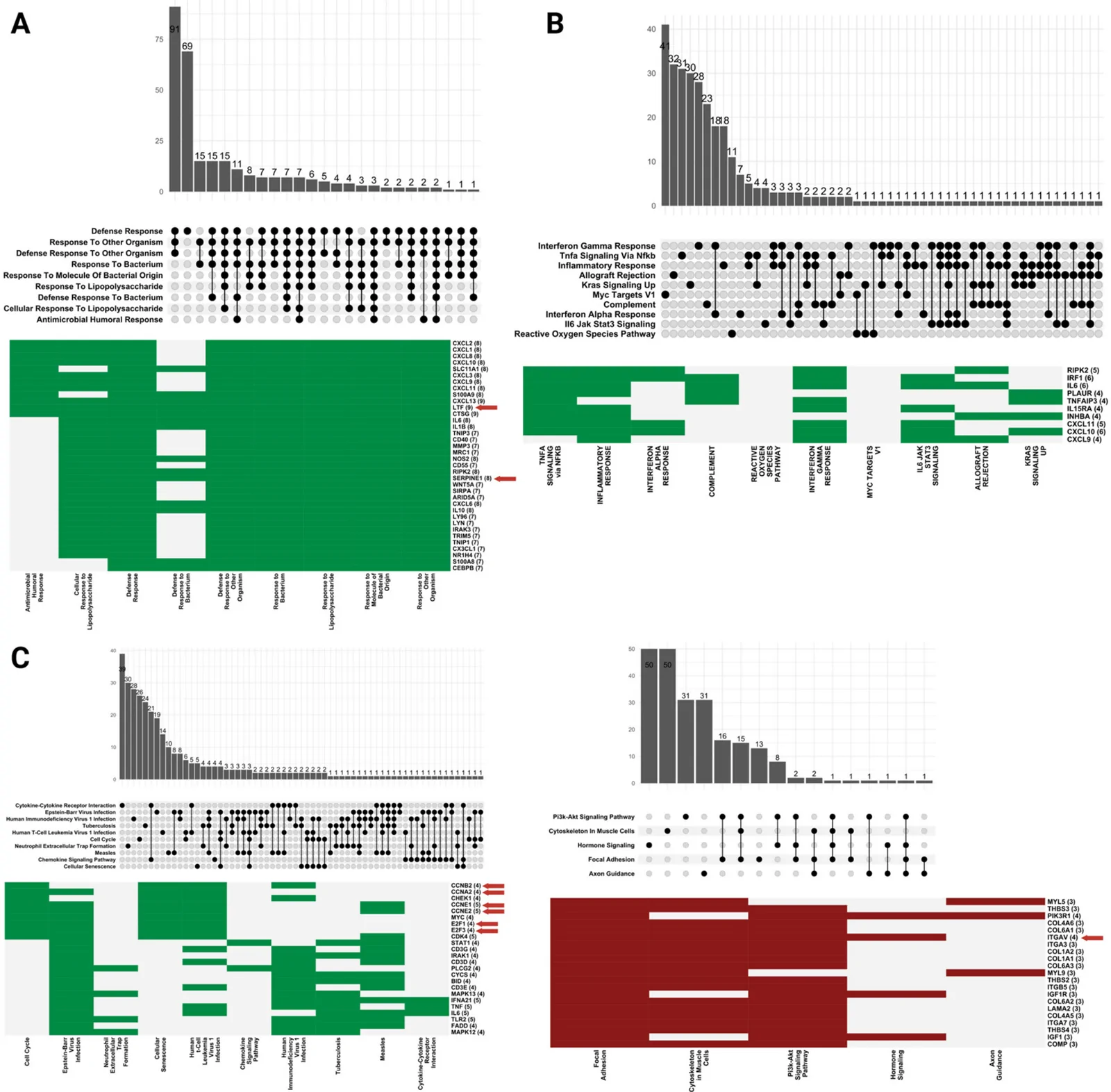
Leading-edge genes in Gene Set Enrichment Analysis. (**A**) UpSet plot (**top**) showing the number of overlapping leading edge genes across the bacteria-centred positively enriched Gene Ontology terms, and the most commonly overlapping genes across those terms (**bottom**). Red arrows indicate genes of interest. (**B**) UpSet plot (**top**) showing the number of overlapping leading edge genes across the positively enriched Molecular Signature Database gene sets, and the most commonly overlapping genes across those gene sets (**bottom**). (**C**) UpSet plots (**top**) showing the number of overlapping leading edge genes across the positively enriched (**left**) and negatively enriched (**right**) Kyoto Encyclopaedia of Genes and Genomes pathways, and the most commonly overlapping genes across those pathways (bottom left and right, respectively). Red arrows indicate genes of interest.

Finally, KEGG was used to investigate the differences in higher-level cellular pathways seen in the *F. nucleatum*+ group. Figure 3E shows the top ten activated KEGG pathways within the group. Multiple activated pathways followed infection and immunity trends, while some of the suppressed pathways were within themes of signalling and regeneration. Several genes were shown to overlap between the positively enriched MSigDb gene sets as well as the various KEGG pathways, indicating proliferative and immune-centred biology (Figure A4).

## 3. Discussion

Bacterial detection has long been at the forefront of medical diagnosis, and methods to identify bacterial pathogens have evolved from classification by metabolites or cell wall structure to identification by sequencing techniques [12]. This exploratory study used a targeted sequencing technique, TempO-Seq™, to identify *F. nucleatum* sequences within human FFPE tissue. This allowed for the subsequent genomic and transcriptomic analysis of *F. nucleatum*+ and *F. nucleatum*- patients, which revealed distinct profiles that could affect the way colorectal adenomas develop and behave.

TempO-Seq™ works by targeting a specific region in every gene and does so directly from lysates prepared from a variety of starting materials [13], circumventing issues with library preparation and data storage, while avoiding the need for RNA extraction from FFPE tissue, making it a useful non-discovery tool for targeted applications. Previous work demonstrated that TempO-Seq™ is as sensitive in fresh lysates as it is in lysates from fixed tissue, and that the output quality was not affected by tissue age or fixation time [14]. Turnbull et al. (2020) [15] concluded that for FFPE tissue, TempO-Seq™ was among the best new platforms for expression profile generation.

Results for the detection of *F. nucleatum* using TempO-Seq™ are presented here, where bespoke probes were considered sensitive and specific and were able to detect their target in FFPE polyp tissue. Quality-check experiments demonstrated that the newly designed *F. nucleatum* probes were able to exclusively detect their target in samples where purified *F. nucleatum* RNA was available, suggesting their specificity. Furthermore, they were sensitive to low quantities of their target RNA, as evidenced by the signal observed in samples where *F. nucleatum* RNA was diluted in human RNA. This level of sensitivity is crucial, as research on CRC tissue using other techniques suggests that no more than 13% of samples test positive for *F. nucleatum* [7,16]. Interestingly, this was comparable to the percentage of *F. nucleatum*+ patients within the tested INCISE subset (11%).

The descriptive analysis of the mutational landscape of the *F. nucleatum*+ and *F. nucleatum*- patients revealed some interesting results. *KRAS* was not among the top 10 most mutated genes in *F. nucleatum*+ samples. This is contrary to previous studies that reported enrichment of *F. nucleatum* in *KRAS*-mutant CRC [17,18]. However, this could point to a major difference between colorectal adenomas and CRC, and although it is the result of a very small exploratory sample size, it warrants further investigation and validation in a larger sample group. There were mutations in immune-related genes, which allude to the possibility of an altered immune response, while the three overlapping genes, *APC*, *HLA-A*, and *ZFHX3*, suggested an *F. nucleatum*-independent, host-driven mutational pattern of these genes.

Furthermore, host transcriptomic analysis identified DEGs in the *F. nucleatum*+ group. Of the upregulated genes, *CXCL1* and *CXCL2*, both members of the CXC chemokine subfamily, were in the top ten most upregulated genes and they function as neutrophil chemoattractants involved in pro-tumour inflammation [19]. SOD2 was linked to neutrophil infiltration and CRC progression [20], and PSMA7 was shown to directly inhibit NOD2, which functions to recognise bacteria and induce the release of relevant pro-inflammatory cytokines [21]. RNF114 has been shown to be a T-cell regulator [22], and a promoter of cell proliferation and stemness in CRC cell line experiments [23].

Further insights were gained into the nature of this immune-dominated transcriptomic profile from GO GSEA, as nine microbial terms relating to bacterial response and defence were positively enriched. *LTF*, the gene encoding lactoferrin, was shared across all nine terms. Lactoferrin is an important anti-microbial protein that defends against bacteria by a variety of mechanisms, such as chelating iron to deprive bacteria of its growth-restoring potential [24].

Another commonly shared leading-edge gene between these terms, *SERPINE1*, has been shown to be protective against Gram-negative pneumonia infections [25], supporting the results presented here, where it is shared across anti-bacterial and defence terms. However, *SERPINE1* has been shown to promote CRC through Notch activation [26]. While *SERPINE1* could be acting in a protective capacity against *F. nucleatum* in adenoma tissue, this could lead to poor outcomes should the adenoma progress into malignancy, suggesting that the presence of *F. nucleatum* could be a poor prognostic factor in adenomas due to the response it elicits.

An interesting link was observed between MSigDb Hallmarks and KEGG GSEA. Positively enriched was the “Reactive Oxygen Species (ROS) Pathway” gene set in MSigDb Hallmarks GSEA and the “Neutrophil Extracellular Traps (NETs) Formation” pathway in KEGG. First described by Brinkmann et al. (2004) [27], NETs resemble webs and are protective structures that capture and kill pathogens. NETs form due to many different stimuli, one of which is induction by ROS [28]. *F. nucleatum* has been shown to induce ROS production in CRC cell line experiments [29], and NETs were shown to be formed during sepsis through LPS from bacteria binding to TLR4, which leads to the activation of platelets as they associate with neutrophils [30]. While an *F. nucleatum*/ROS/NETs formation axis has been reported for CRC [31], these results suggest an earlier involvement for *F. nucleatum* in ROS-induced NETosis.

The upregulation of the replication fork regulator *RTF2* and downregulation of the DNA methyltransferase *DMAP1* and the lipid kinase *AGK* suggest a proliferative signature in *F. nucleatum*+ patients. *RTF2* is involved in cell growth and replication stress [32], and both DMAP1—involved in DNA repair and stability [33]—and *AGK*—involved in homeostasis regulation—have tumour-suppressing functions in cancer [34,35]. The differential expression of these genes supports the proliferative signature highlighted in GSEA from both MSigDb Hallmarks and KEGG. *F. nucleatum* can influence cell signalling pathways involved in cell proliferation [36,37]. This was supported by the positive enrichment of cell cycle regulatory genes such as *CDK4* and *MYC*, cyclin-encoding genes *CCNA2*, *CCNB2*, *CCNE1*, and *CCNE2*, as well as transcription factors *E2F1* and *E2F3*, all of which are involved in the decision to enter and commit to the cell cycle, and have been shown to be mutated in CRC [38].

*CAPN9* was downregulated in the *F. nucleatum*+ patients. A calpain involved in gastric injury defence [39], *CAPN9* was a leading-edge gene in the negatively enriched gene set from MSigDb Hallmarks. Furthermore, negatively enriched KEGG pathways relating to cellular structure and localisation offer insights into the relationship between *F. nucleatum* and the eventual migration of cells from the colorectal epithelium to distant sites. Although *F. nucleatum* was found in liver metastasis from CRC primaries [40], and integrins such as *ITGAV* were associated with poor CRC survival [41], the negative enrichment observed here may suggest that the migratory effects reported in *F. nucleatum*+ CRC patients develop with progression to malignancy.

This work, while exploratory in nature due to the small sample size, provided evidence of the possibility of using novel techniques to detect and visualise *F. nucleatum* in archival tissue of colorectal adenomas, and while the findings presented here are preliminary and must be validated in a larger cohort, TempO-Seq™ offered near-adequate specific and sensitive detection of *F. nucleatum*. Despite the small sample size and group imbalance between the *F. nucleatum*+ and *F. nucleatum*- patients, the transcriptomic profile of *F. nucleatum*+ patients suggests a possible role for *F. nucleatum* in preparing and supporting a proliferative, inflamed environment in the adenoma stage. Future work should focus on validating these findings in larger datasets, as well as delving into the mechanisms behind *F. nucleatum* and immunity in colorectal adenomas, as these results could pave the way for improved prediction of metachronous adenomas or CRC to improve clinical outcomes.

## 4. Materials and Methods

### 4.1. Patient Cohort

Patients from the Integrated Technologies for Improved Polyp Surveillance (INCISE; Johnstone et al., 2021 [42]) cohort were randomly selected to build an unbiased, randomly selected sub-cohort consisting of 198 bowel screening patients from the Greater Glasgow and Clyde area (2009–2016) who on index colonoscopy, had a minimum of one polyp removed and received follow-up colonoscopies as part of the Scottish Surveillance programme for 6 months—6 years after the index colonoscopy. Pathological information on each excised polyp was obtained from histopathology reports. These included but were not limited to the number of polyps present at index and follow-up colonoscopies, polyp histology (whether a polyp presented adenoma or serrated features), polyp morphology (characterised by the presence or absence of villous features), polyp location (right/left colon, or rectum), polyp size (<10 mm≤), and presence of advanced polyps. From these clinical variables, the BSG2020 Guidelines were determined, and the risk category was recorded for each patient. Patient status at follow-up surveillance colonoscopy was dependent on the presence or absence of metachronous polyp(s) or CRC and was determined using electronic endoscopy reporting software (Ver. 2.5, Unisoft GI Reporting Software, Unisoft Medical Systems, London, UK) and the Telepath Laboratory Information Management System electronic pathology database. All analyses were done on the index polyp from each patient, defined as the largest polyp in patients with multiple polyps or the only polyp in patients with single polyps.

### 4.2. Human and Bacterial RNA Preparation and Titration

Human qPCR Reference Total RNA (URR; Cat. 636690) and human Brain Total RNA (BRR; Cat. 636530) were purchased from Takara Bio. (Shiga, Japan). These were diluted in water to a concentration of 100 ng/µL, then aliquoted and stored at −80 °C until required. *F. nucleatum* (ATCC 10953) cultures were kindly provided by Prof. Gordon Ramage (University of Glasgow). RNA extraction was done using RNeasy^®^ Mini Kit (Cat. 74104, Qiagen, Hilden, Germany) with no deviation from the manufacturer’s instructions. Human URR was prepared to a final concentration of 10 ng/μL by adding 12 μL of URR to 108 μL of UltraPure water. This was used to prepare 10-fold serial dilutions of bacterial RNA to human RNA, as shown in Table A1.

### 4.3. TempO-Seq™

#### 4.3.1. Bacterial Probe Design and In Silico Quality Check

*F. nucleatum* subsp. *Polymorphum* (ATCC10953) 16S ribosomal RNA (NCBI Ref. NR_117288.1) was used to compare against prospective probe sequences. Sequences found in the literature (e.g., Yang et al., 2016 [36]; Nishimura et al., 2021 [43]) were used as a starting point for the design of the *F. nucleatum* probes. To identify the potential target regions and specificity to *F. nucleatum*, the probe sequences were run on the SILVA ribosomal RNA database project platform [44,45]. Probe sequences were entered in the 5′–3′ direction, and 0 mismatches were allowed before the TestProbe3.0 function was run. A table of matched regions was exported as a “.csv” file and manually checked for compatibility with organisms and number of mismatches. Reverse-complementary sequences to the probes were obtained from a reverse-complement online tool [46]. These were checked against the 16S region for *F. nucleatum* to ensure target binding and to check the sequences flanking the target. Sequences targeted by probes had to be at least 50 base pairs long. The eight potential Fusobacterium probe sequences were checked for organism specificity, GC content (40–60%), melting temperature (60–70 °C), presence of homopolymers, and self-annealing capacity and hairpin structure formation. A summary of the probe sequences and quality control parameters is shown in Table A2. Human and bacterial probes that passed in silico quality checks were ordered for manufacture by IDT through their LabReady service at a concentration of 100 μM in IDTE buffer at pH8 (Table 2).

#### 4.3.2. Human Probe Design and In Silico Quality Check

Human probes were selected as internal assay controls. These included SOX9, E-Cadherin, β-Catenin, COX2, and p53. Probe sequences were already available on BioClavis databases and were tested in silico using the UCSC BLAT tool (v. 39x1) [47] for target specificity in human genomes.

#### 4.3.3. Probe Pooling

Detector oligo (DO) pools of the human probes and bacterial probes were prepared as outlined in Table A3 and Table A4. Each downstream DO (DDO) and upstream DO (UDO) of a probe pair was added at the same volume and concentration.

#### 4.3.4. TempO-Seq™ Assay

Unless specified otherwise, all reagents used in the Templated Oligo Assay with Sequence Readout (TempO-Seq™) were supplied by BioSpyder (Carlsbad, CA, USA). TempO-Seq™ was run according to the manufacturer’s instructions. In brief, healthy colon tissue sections (Product code: HP-311) obtained from Amsbio (Cambridge, UK) and INCISE tissue sections prepared by GTRF were sectioned to 4 μm thick and lysed using 2 μL Lysis Buffer/1 mm^2^. The tissue was deparaffinised by incubating at 95 °C for 5 min. Further processing included a 30 min incubation at 37 °C in 2.5% FFPE Protease, followed by the addition of the DOs for hybridisation. Hybridisation was performed at 70 °C for 10 min, followed by a controlled temperature drop to 45 °C at a rate of 0.5 °C/min. The assay plates were held at 45 °C for 16 h, and the plates were cooled to 25 °C. Excess DOs were removed by nuclease digestion, and hybridised DDO and UDO probe pairs were ligated together prior to PCR amplification, which allowed the samples to be indexed and pooled into a sequencing run. Resulting TempO-Seq™ libraries were pooled into a sequencing library prior to sequencing by Illumina MiniSeq Sequencing System (Illumina, San Diego, CA, USA). Sequencing reads were demultiplexed by BCL2FASTQ software (v2.20.0, Illumina, San Diego, CA, USA), and raw counts per gene per sample were generated. All experiments included BRR and URR controls, at least one dilution of *F. nucleatum* in human RNA, and no sample controls (NSCs) in the same 96-well plate, all run in duplicate.

#### 4.3.5. Analysis of TempO-Seq™ Read Counts

Sums of read counts from each probe for each sample were averaged, and then normalised by dividing the read count value by the product of the sum of read counts for each probe and the average of probe sums of all samples, as shown in the equation below:

$$\frac{Read\;Count\;Value\;of\;Each\;Probe}{\left(Sum\;of\;Read\;Counts\;for\;Each\;Probe\times Average\;of\;Probe\;Sums\;of\;All\;Samples\right)}$$

Thresholding of *F. nucleatum* negative or positive status was determined by the *F. nucleatum* probe signal on the human reference RNA (BRR and URR). The average plus 1 standard deviation for each *F. nucleatum* probe for those samples was calculated, and any signal from the *F. nucleatum* probes on the INCISE polyp lysates above the thresholds was considered an *F. nucleatum* positive sample for that probe. This analysis was done on MS Excel (Ver. 2301, Microsoft, Redmond, WA, USA).

### 4.4. Validation of TempO-Seq™ Using RNAscope^®^

RNAscope^®^ in situ hybridisation was performed as an independent orthogonal assay. Patient tissue was requested from NHS Greater Glasgow and Clyde Tissue and cut by GTRF, no more than 2 weeks prior to use. Slides were stored at 4 °C until used. RNAscope^®^ 2.5 LSx Reagent Kit–BROWN (Ref. 322700, ACD-biotehne, Abingdon, UK) was used according to the manufacturer’s instructions and run overnight according to the BOND Polymer Refine Detection system (Ref. DS9800, Leica Biosystems, Newcastle upon Tyne, UK) protocol. Three levels of experimental probing were performed, in addition to assay positive and negative controls. The RNAscope^®^ 2.5 LS Positive Control Probe–Hs-UBC (Ref.312028, ACD-biotehne, Abingdon, UK) was used to detect the presence of intact RNA by targeting *Homo sapiens* Ubiquitin C, the RNAscope^®^ 2.5 LS Probe–EB-16S-rRNA probe (Ref. 464468, ACD-biotechne, Abingdon, UK) was used to detect bacterial RNA by targeting *Eubacteria* 16S rRNA, and RNAscope^®^ 2.5 LS Probe–B-Fusobacterium-23S-1zz (Ref. 486408, ACD-biotechne, Abingdon, UK) was used to detect *F. nucleatum* RNA by targeting *F. nucleatum* 23S rRNA. RNAscope^®^ 2.5 LS Probe–Hs-TBP (Ref. 314298, ACD-biotechne, Abingdon, UK) was used as a positive control targeting *Homo sapiens* TATA-Box Binding Protein, and RNAscope^®^ 2.5 LS Negative Control Probe–DapB (Ref. 312038, ACD-biotechne, Abingdon, UK) was used as a negative control targeting *Bacillus subtilis* dihydrodipicolinate reductase.

The slides were dehydrated manually by placing them in water, and then in 70% ethanol for 2 min, followed by 2 times in 100% ethanol for 2 min each, and finally in xylene twice for 2 min each. Slides were mounted with coverslips using Pertex mounting medium (Ref. 00801-EX, HistoLab, Gothenburg, Sweden), which was allowed to dry for a minimum of 1 h. They were sent to the Beatson Institute Histology Services for scanning at 40×.

### 4.5. Mutational Profiling

Sample DNA sequencing and variant calling were performed by the Glasgow Genomic Innovation Alliance (Glasgow, UK) using the Agilent SureSelect XT2 HS2 method (Agilent, Santa Clara, CA, USA), and mutational analysis was performed on RStudio (2023.12.0+369; Boston, MA, USA) using the Maftools (Ver. 2.18.0; Mayakonda et al., 2018 [48]) package under the BiocManager Repository (Morgan & Ramos, 2023 [49]; Ver. 1.30.22) as previously described in Al-Badran et al., (2024) [50].

### 4.6. Bulk Transcriptomics

Bulk transcriptomics for 198 patients were processed and analysed as previously described in Al-Badran et al. (2024) [50]. In brief, all transcriptomics data originated from TempO-Seq™ whole-transcriptome profiling experiments in the INCISE cohort. Probe variance across all samples was calculated using the sapply function. Probes with a variance less than the 25th percentile were removed. Samples with a low read count (<95% CI [<2.28 × 10^6^ counts]) were identified with stats (Ver. 4.2.3) and removed, as well as samples which did not match batch information. The counts were batch-corrected using ComBat_seq (Zhang, Parmigiani & Johnson, 2020 [51,52]; sva Ver. 3.46.0). Probe IDs were mapped to gene symbols, and duplicated genes were collapsed using MaxMeans (Langfelder & Horvath, 2008, 2012 [53,54]; WGCNA Ver. 1.72-1), resulting in the remaining 14,993 genes. Normalised counts were generated using quantile normalisation and a log2 +1 transformation.

Differentially expressed genes (DEGs) were identified using DESeq2 (Love, Huber & Anders, 2014 [55]; Ver. 1.42.1), which allowed for the extraction of Log2 Fold-Change, *p*-value, and adjusted *p*-value (*p*Adj).

Gene set enrichment analysis (GSEA) was used to compare gene expression within predefined biological pathways between two groups, using differential expression data containing log2 fold changes generated from DESeq2. GSEA was performed using ClusterProfiler (Ver.3.18; Yu et al., 2012 [56]) and the *Homo sapiens* organism annotation. Three different ontologies/pathway databases were explored: Gene Ontology (GO; Ashburner et al., 2000 [57]; Consortium et al., 2023 [58]), Molecular Signature Database (MSigDb; Subramanian et al., 2005 [59]; Liberzon et al., 2011, 2015 [60,61]), and the Kyoto Encyclopaedia of Genes and Genomes (KEGG; Kanehisa & Goto, 2000 [62]; Kanehisa, 2019 [63]; Kanehisa et al., 2024 [64]).

All data were visualised using ggplot2 (Ver. 3.5.0; Wickham, 2016 [65]). geom_point() was used to visualise volcano plots to display DEGs. ComplexHeatmap (Ver. 2.21.1; Gu, 2022 [66]) was used to generate heatmaps to investigate any patterns of expression of DEGs between the two groups. For GSEA, bar plots were generated using geom_col to show statistically significant upregulated and downregulated gene sets ranked by normalised enrichment scores (NES).

## Figures and Tables

**Table 1:** Summary of Thresholds for *F. nucleatum* TempO-Seq™ Probes.

	Magenta Probe	Cyan Probe	Gold Probe
			
Threshold	1572.237	3104.139	39.9422
Detection Rate per Probe, n (%)	17 (9%)	47 (24%)	21 (11%)

**Table 2:** Identification Guide of *F. nucleatum* Probes.

Probe ID	Probe Name	
Fn16S_113694	Green	
Fn16S_113695	Indigo	
Fn16S_113696	Magenta	
Fn16S_113697	Cyan	
Fn16S_113698	Gold	

## Data Availability

The datasets generated and/or analysed during the current study are included with the larger INCISE datasets available in the EMBL-EBI repository ArrayExpress through accession numbers E-MTAB-15346 and E-MTAB-15349.

## Abbreviations

The following abbreviations are used in this manuscript:

CRC	Colorectal Cancer
FFPE	Formalin-fixed Paraffin-embedded
BSG	British Society of Gastroenterology
URR	Human Reference Total RNA
BRR	Human Brain Total RNA
DO	Detector Oligo
DDO	Downstream Detector Oligo
UDO	Upstream Detector Oligo
TempO-Seq™	Templated Oligo Sequencing
GTRF	Glasgow Tissue Research Facility
NSC	No Sample Controls
DEGs	Differentially Expressed Genes
GSEA	Gene Set Enrichment Analysis
GO	Gene Ontology
MSigDB	Molecular Signature Database
KEGG	Kyoto Encyclopaedia of Genes and Genomes
NES	Normalised Enrichment Score
pAdj	Adjusted *p*-value
ROS	Reactive Oxygen Species
NETs	Neutrophil Extracellular Traps

## Appendix A

**Table A1:** 10-Fold Serial Dilutions of Bacterial RNA in Human RNA.

Dilution	*F. nucleatum* RNA	Human URR
Fn1:Hs1	6 μL purified RNA at 10 ng/µL	6 μL at 10 ng/μL
Fn1:Hs10	2 μL purified RNA at 10 ng/µL	18 μL at 10 ng/μL
Fn1:Hs100	2 μL from Fn1:Hs10 at 10 ng/µL	18 μL at 10 ng/μL
Fn1:Hs1000	2 μL from Fn1:Hs100 at 10 ng/µL	18 μL at 10 ng/μL
Fn1:Hs10,000	2 μL from Fn1:Hs1000 at 10 ng/µL	18 μL at 10 ng/μL
Fn1:Hs100,000	2 μL from Fn1:Hs10,000 at 10 ng/µL	18 μL at 10 ng/μL
Fn1:Hs1,000,000	2 μL from Fn1:Hs100,000 at 10 ng/µL	18 μL at 10 ng/μL

URR: Universal Reference RNA. Fn: *F. nucleatum*. Hs: *Homo sapiens*.

**Table A2:** Parameters of *F. nucleatum* Probe Quality Check.

	Probe ID	Probe Name	Direction		Sequences (5′–3′)	Organism Specificity (SILVA)	GC Content (%)	Tm (°C)	Homopolymers	Self-Annealing Capacity (Bases)	Hairpin Probability (%)
Probe 1	Fn16S_113694	Green		DDO	TCTAGTTACACAGTTTCCAACGCAA	*Fusobacterium*	40	59.7	No	4	<50
				UDO	TACAGAGTTGAGCCCTGCATTTTCA	*Fusobacterium*	44	61.3	Yes TTTT	4	70–80
Probe 2	Fn16S_113695	Indigo		DDO	ACGAATTTCACCTCTACACTTGTAG	*Fusobacterium*	40	59.7	No	10	80–90
				UDO	TTCCGCTTACCTCTCCAGTACTCTA	*Fusobacterium* and uncultured rumen bacterium	48	63	No	8	<50
Probe 3	Fn16S_113696	Magenta		DDO	TACCTCTCCAGTACTCTAGTTACAC	*Fusobacterium*	44	61.3	No	12	<50
				UDO	AGTTTCCAACGCAATACAGAGTTGA	*Fusobacterium*	40	59.7	No	12	80–90
Probe 4	Fn16S_113697	Cyan		DDO	GTCCCCGTCAATTCCTTTGAGTTTC	Rice and marine metagenome	48	63	Yes CCCC	8	60–70
				UDO	ATACTTGCGTGCGTACTCCCCAGGC	Uncultured *Clostridiales bacterium*	60	67.9	Yes CCCC	6	50–60
Probe 5	Fn16S_113698	Gold		DDO	ACCTCTCCAACTAGCTAATGGGACG	*Fusobacterium* and uncultured *Treponema* sp.	54	64.6	No	14	<50
				UDO	CAAAGCTCTCTCACAGCGCATGTAG	*Fusobacterium* and uncultured organism	52	64.6	No	8	50–60
Probe 6	NA	NA		DDO	TCCCCGTCAATTCCTTTGAGTTTCA	Rice and marine metagenome	44	61.3	Yes CCCC	8	60–70
				UDO	TACTTGCGTGCGTACTCCCCAGGCG	Uncultured bacteria	64	69.5	Yes CCCC	6	90–95
Probe 7	NA	NA		DDO	GCCGTTACCTCTCCAACTAGCTAAT	*Fusobacterium* and others	48	63 °C	No	8	<50
				UDO	GGGACGCAAAGCTCTCTCACAGCGC	*Fusobacterium*	64	69.5	No	4	60–70
Probe 8	NA	NA		DDO	TAGTTACACAGTTTCCAACGCAATA	*Fusobacterium*	36	58.1	No	4	<50
				UDO	CAGAGTTGAGCCCTGCATTTTCACA	*Fusobacterium*	48	63	Yes TTTT	6	<50

Probe directions and sequences are included. Probes specific to *Fusobacterium* are those with a GC content between 40 and 60%, melting temperatures between 60 and 70 °C, a low self-annealing capacity, homopolymers less than 6-mers in length (preferably none), and a hairpin probability <90%. Specific probes that were sent for manufacture were assigned Probe IDs. Failed probes that were not manufactured were not assigned IDs (NA). Probes were given colour-coded names for simplicity. Probe: pair of detector oligos, DDO: Downstream Detector Oligo, UDO: Upstream Detector Oligo, GC Content: Guanine-Cytosine Content, and Tm: Melting Temperature.

**Table A3:** Components, Volumes, and Concentrations of Primary Detector Oligo Pools.

DO Pool	Components	Components Volume and Concentration		Notes
		Each Oligo	Oligo Pool (Total)	
DO1	DDO_Fn16S_113694	5 μL at 100 μM	50 μL of oligos at 10 μM	DO Pool 1 includes all the Fuso probes only.
	UDO_Fn16S_113694	5 μL at 100 μM		
	DDO_Fn16S_113695	5 μL at 100 μM		
	UDO_Fn16S_113695	5 μL at 100 μM		
	DDO_Fn16S_113696	5 μL at 100 μM		
	UDO_Fn16S_113696	5 μL at 100 μM		
	DDO_Fn16S_113697	5 μL at 100 μM		
	UDO_Fn16S_113697	5 μL at 100 μM		
	DDO_Fn16S_113698	5 μL at 100 μM		
	UDO_Fn16S_113698	5 μL at 100 μM		
DO2	DDO_SOX9_22075	5 μL at 100 μM	100 μL of oligos at 5 μM	DO Pool 2 includes all the human probes and all the Fuso probes.
	UDO_SOX9_22075	5 μL at 100 μM		
	DDO_COX2_1514	5 μL at 100 μM		
	UDO_COX2_1514	5 μL at 100 μM		
	DDO_TP53_7287	5 μL at 100 μM		
	UDO_TP53_7287	5 μL at 100 μM		
	DDO_CDH1_1186	5 μL at 100 μM		
	UDO_CDH1_1186	5 μL at 100 μM		
	DDO_CTNNB1_1633	5 μL at 100 μM		
	UDO_CTNNB1_1633	5 μL at 100 μM		
	DDO_Fn16S_113694	5 μL at 100 μM		
	UDO_Fn16S_113694	5 μL at 100 μM		
	DDO_Fn16S_113695	5 μL at 100 μM		
	UDO_Fn16S_113695	5 μL at 100 μM		
	DDO_Fn16S_113696	5 μL at 100 μM		
	UDO_Fn16S_113696	5 μL at 100 μM		
	DDO_Fn16S_113697	5 μL at 100 μM		
	UDO_Fn16S_113697	5 μL at 100 μM		
	DDO_Fn16S_113698	5 μL at 100 μM		
	UDO_Fn16S_113698	5 μL at 100 μM		
DO3	DDO_SOX9_22075	5 μL at 100 μM	80 μL of oligos at 6.25 μM	DO Pool 3 comprises the same elements as DO Pool 2 at a different final concentration.
	UDO_SOX9_22075	5 μL at 100 μM		
	DDO_COX2_1514	5 μL at 100 μM		
	UDO_COX2_1514	5 μL at 100 μM		
	DDO_TP53_7287	5 μL at 100 μM		
	UDO_TP53_7287	5 μL at 100 μM		
	DDO_CDH1_1186	5 μL at 100 μM		
	UDO_CDH1_1186	5 μL at 100 μM		
	DDO_CTNNB1_1633	5 μL at 100 μM		
	UDO_CTNNB1_1633	5 μL at 100 μM		
	DDO_Fn16S_113696	5 μL at 100 μM		
	UDO_Fn16S_113696	5 μL at 100 μM		
	DDO_Fn16S_113697	5 μL at 100 μM		
	UDO_Fn16S_113697	5 μL at 100 μM		
	DDO_Fn16S_113698	5 μL at 100 μM		
	UDO_Fn16S_113698	5 μL at 100 μM		
DO4	DDO_SOX9_22075	5 μL at 100 μM	70 μL of oligos at 7.14 μM	DO Pool 4 excludes COX2 but includes the remaining human probes and bacterial probes. This pool was used in different titrations with DO5 to make up DOs 6–10.
	UDO_SOX9_22075	5 μL at 100 μM		
	DDO_TP53_7287	5 μL at 100 μM		
	UDO_TP53_7287	5 μL at 100 μM		
	DDO_CDH1_1186	5 μL at 100 μM		
	UDO_CDH1_1186	5 μL at 100 μM		
	DDO_CTNNB1_1633	5 μL at 100 μM		
	UDO_CTNNB1_1633	5 μL at 100 μM		
	DDO_Fn16S_113696	5 μL at 100 μM		
	UDO_Fn16S_113696	5 μL at 100 μM		
	DDO_Fn16S_113697	5 μL at 100 μM		
	UDO_Fn16S_113697	5 μL at 100 μM		
	DDO_Fn16S_113698	5 μL at 100 μM		
	UDO_Fn16S_113698	5 μL at 100 μM		
DO5	DDO_COX2_1514	5 μL at 100 μM	10 μL at 50 μM	DO Pool 5 includes COX2 only. It was used with DO4 in different titrations to make up DOs 6–10.
	UDO_COX2_1514	5 μL at 100 μM		

DO: Detector Oligo, UDO: Upstream detector oligo, DDO: Downstream detector oligo.

**Table A4:** Composition of DO Pools as Used Experimentally.

DO Pool	Stock Concentration	Final Concentration	Composition	
			DOs	TE Buffer
DO1	10 μM	30 nM	3 μL of 10 μM DO1	997 μL
DO1	30 nM	0.3 nM	5 μL of 30 nM DO1	495 μL
DO2	5 μM	30 nM	3 μL of 5 μM DO2	497 μL
DO2	30 nM	0.3 nM	5 μL of 30 nM DO2	495 μL
DO3	6.25 μM	30 nM	3 μL of 6.25 μM	622 μL
DO3	30 nM	0.3 nM	5 μL of 30 nM	495 μL
DO4	7.14 μM	300 nM	5 μL of 7.14 μM DO4	114.05 μL
DO4	300 nM	30 nM	20 μL of 30 nM DO4	180 μL
DO5	50 μM	300 nM	5 μL of 50 μM DO5	828.33 μL
DO5	300 nM	30 nM	20 μL of 300 nM DO5	180 μL
DO5	30 nM	3 nM	20 μL of 30 nM DO5	180 μL
DO9	300 nM DO4 3 nM DO5	3 nM DO4 0.03 nM DO5	5 μL of 300 nM DO4 5 μL of 3 nM DO5	490 μL

**Table A5:** Relationship between *F. nucleatum* Status and Clinical Characteristics in the INCISE Cohort Subset with TempO-Seq™ based *F. nucleatum* Status.

		*F. nucleatum* Status (n = 198)				
		Negative		Positive		*p*
		176	89%	22	11%	
Sex	Female	57	32%	7	32%	0.957
	Male	119	68%	15	68%	
Age	50–74 (Screening Age)	167	95%	21	96%	0.909
	75+ (Above Screening Age)	9	5%	1	4%	
Site	Right Colon	23	13%	4	18%	0.734
	Left Colon	129	74%	16	73%	
	Rectum	23	13%	2	9%	
High Grade Dysplasia	Absent	146	83%	15	68%	0.094
	Present	30	17%	7	32%	
Number of Polyps	1	59	33%	7	32%	0.156
	2–4	82	47%	14	64%	
	5+	35	20%	1	4%	
Polyp Type	Non-adenoma *	6	3%	4	18%	0.003
	Adenoma	170	97%	18	82%	
BSG2020 Guidelines	Low Risk	65	37%	8	36%	0.958
	High Risk	111	63%	14	64%	
Metachronous Polyps or CRC Detection	No	77	44%	12	55%	0.337
	Yes	99	56%	10	45%	

* Non-adenoma refers to all polyps with non-conventional adenoma features. This includes serrated polyps, hyperplastic polyps, etc.

**Table A6:** *F. nucleatum* Status, Clinicopathological Characteristics, and the Detection of Metachronous Polyps of CRC.

		Univariate Cox Regression		
		HR	95% CI	*p*
Sex	Female	1.0	-	-
	Male	0.862	0.700–1.061	0.160
Age	Screening Age	1.0	-	-
	Above Screening Age	0.775	0.316–1.902	0.578
Site	Right Colon	1.0	-	-
	Left Colon	0.722	0.426–1.222	0.225
	Rectum	0.780	0.426–0.384	0.491
High Grade Dysplasia	Absent	1.0	-	-
	Present	0.699	0.416–1.173	0.175
Number of Polyps	1	1.0	-	-
	2–4	1.140	0.731–1.777	0.563
	5+	2.240	1.338–3.749	0.002
Polyp Type	Adenoma	1.0	-	-
	Non-adenoma *	0.926	0.377–2.272	0.867
BSG2020 Guidelines	Low Risk	1.0	-	-
	High Risk	1.427	0.954–2.134	0.084
*F. nucleatum* Status	Negative	1.0	-	-
	Positive	0.805	0.420–1.544	0.515

* Non-adenoma refers to all polyps with non-conventional adenoma features. This includes serrated polyps, hyperplastic polyps, etc.
